# Redox data of ferrocenylcarboxylic acids in dichloromethane and acetonitrile

**DOI:** 10.1016/j.dib.2020.105650

**Published:** 2020-04-29

**Authors:** Pieter J. Swarts, Jeanet Conradie

**Affiliations:** Department of Chemistry, PO Box 339, University of the Free State, Bloemfontein, 9300, South Africa

**Keywords:** Ferrocenyl acids, cyclic voltammetry, oxidation, solvent effect, electronic effect

## Abstract

Redox data obtained from cyclic voltammetry experiments of the Fe^II/III^ oxidation of six ferrocenyl carboxylic acids is presented in this data in brief article. Data is obtained from the cyclic voltammograms at scan rates of two orders of magnitude (0.05 – 5.00 Vs^−1^) using (i) acetonitrile as solvent and tetrabutylammonium hexafluorophosphate as supporting electrolyte and (ii) dichloromethane as solvent and tetrabutylammonium tetrakispentafluorophenylborate, as the electrolyte. Data is reported *versus* the Fe^II/III^ redox couple of ferrocene. For more insight in the reported data, see the related research article “Solvent and substituent effect on Electrochemistry of ferrocenylcarboxylic acids”, published in Journal of Electroanalytical Chemistry [1].

**Specifications Table**

**Table:** 

**Subject**	Chemistry
**Specific subject area**	Electrochemistry
**Type of data**	Table Image Graph Figure
**How data were acquired**	Princeton Applied Research PARSTAT 2273 potentiostat running Powersuite software (Version 2.58).
**Data format**	Raw Analysed
**Parameters for data collection**	Samples were used as synthesized. All the electrochemical experiments were performed in an M Bruan Lab Master SP glove box under a high purity argon atmosphere (H_2_O and O_2_< 10 ppm).
**Description of data collection**	All electrochemical experiments were done in a 2 ml electrochemical cell containing three-electrodes (a glassy carbon working electrode, a Pt auxiliary electrode and a Pt pseudo reference electrode), connected to a Princeton Applied Research PARSTAT 2273 electrochemical analyser. Data obtained were exported to excel for analysis and diagram preparation.
**Data source location**	University of the Free State Bloemfontein South Africa
**Data accessibility**	With the article
**Related research article**	P.J. Swarts, J. Conradie, Solvent and substituent effect on electrochemistry of ferrocenylcarboxylic acids, J. Electroanal. Chem. (2020) 114164. doi:10.1016/j.jelechem.2020.114164.

**Value of the Data**

• This data provides detailed electrochemical data for six ferrocenyl carboxylic acids in both DCM and ACN for scan rates over two orders of magnitude (0.05 – 5.0 Vs^−1^).
• This data illustrates the influence of the solvent used in cyclic voltammetry experiments, on the formal redox potential of Fe of the ferrocenyl group for ferrocenylcarboxylic acids.
• This data illustrates the influence of the solvent on the peak current-voltage separations, ΔE_p_, of the Fe oxidation peak of ferrocenyl carboxylic acids.
• This data illustrates the electronic influence of electron-withdrawing carbonyl group on the iron's oxidation potential, depending on how close the carbonyl group is to the iron.
• Accurate redox potential data of these ferrocenyl (Fc) carboxylic acids are important, since they are used as ligands in organometallic complexes.

## 1 Data

**Fig. 1:**
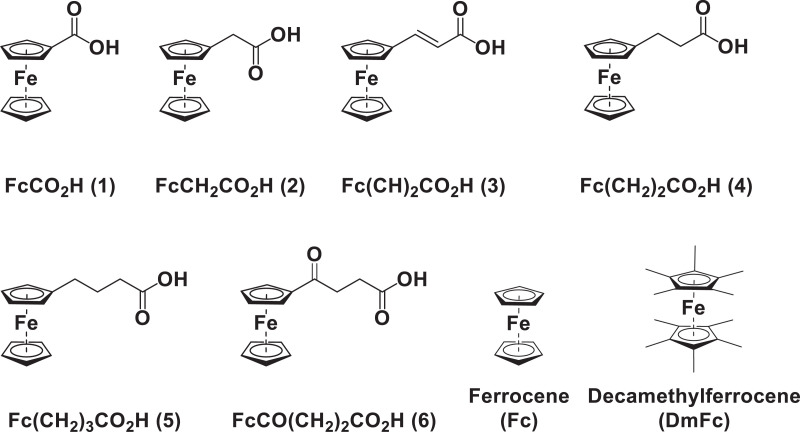
Structure of compounds in this study used for cyclic voltammetry.

**Fig. 2:**
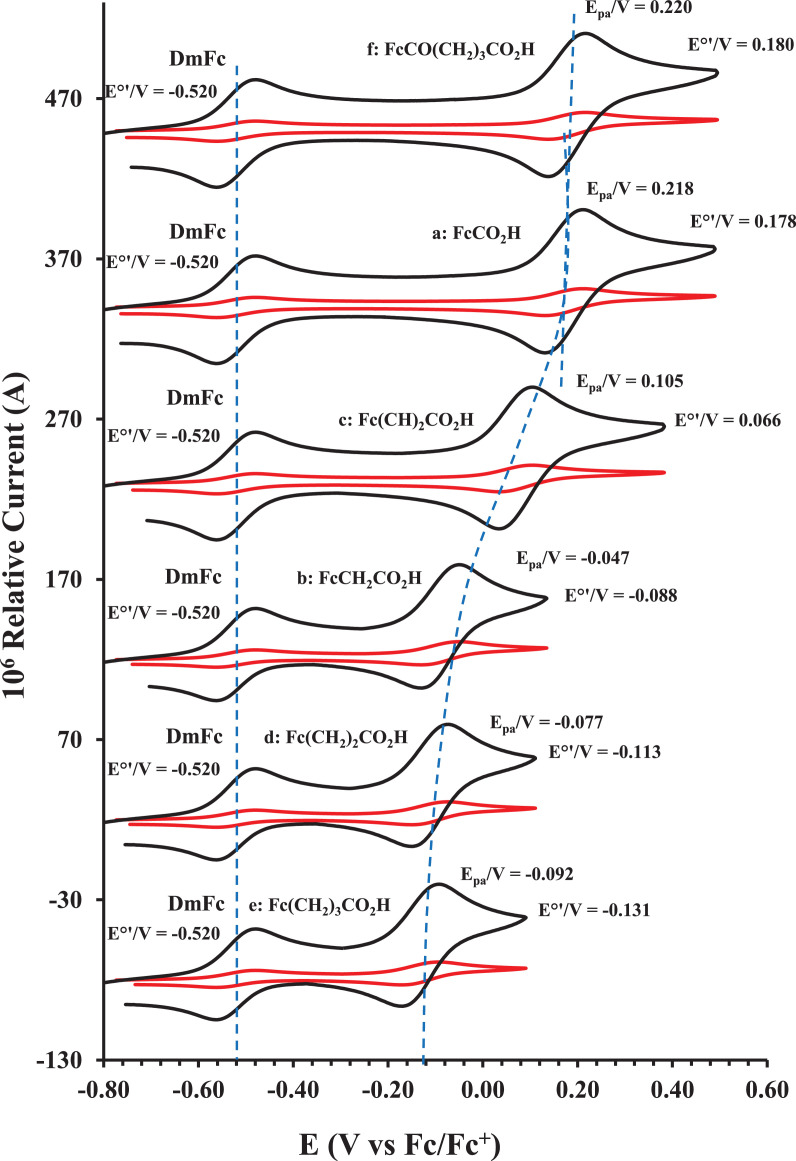
Cyclic voltammogram in ACN of (a) FcCO_2_H, (b) FcCH_2_CO_2_H. (c) Fc(CH)_2_CO_2_H, (d) Fc(CH_2_)_2_CO_2_H, (e) Fc(CH_2_)_3_CO_2_H and (f) FcCO(CH_2_)_2_CO_2_H, at scan rates 0.100 (red) and 5.00 (black) Vs^−1^. Scans initiated in a positive direction. Data for the peak oxidation potential (E_pa_) and the formal reduction potential (E^0’^) of DmFc (internal standard, left) and the indicated ferrocene-containing carboxylic acid (right) are indicated in V.

**Fig. 3:**
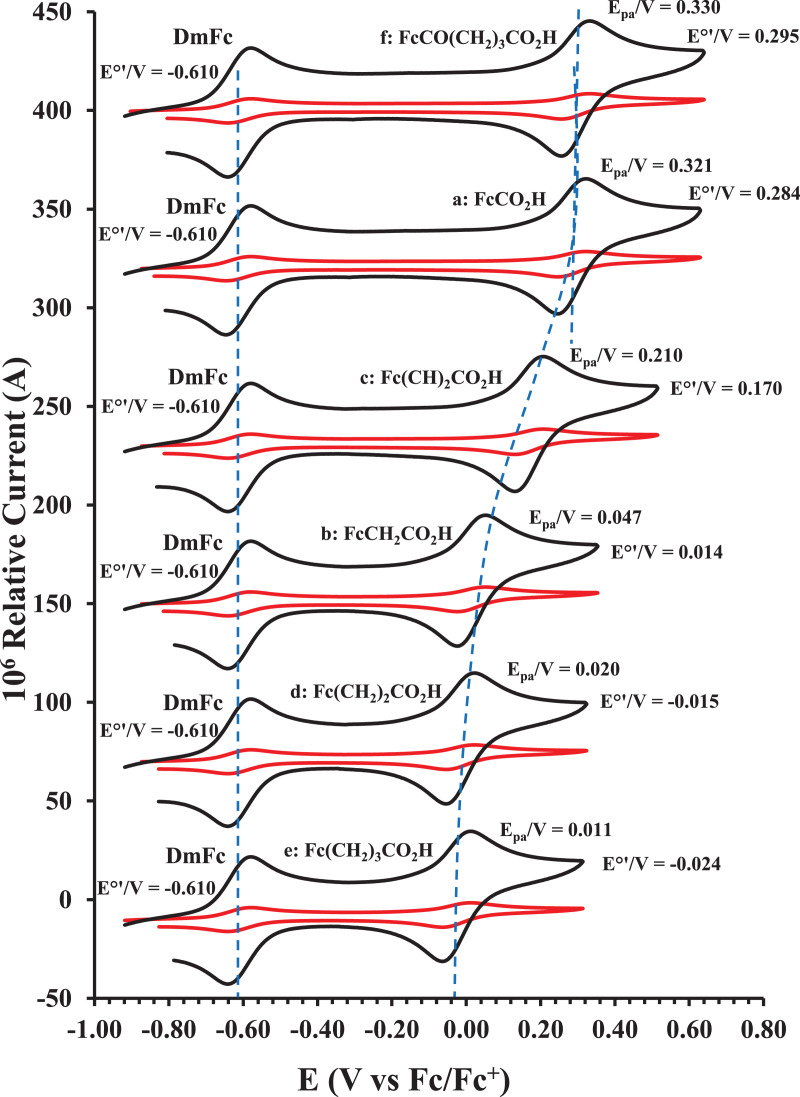
Cyclic voltammogram in DCM of (a) FcCO_2_H, (b) FcCH_2_CO_2_H. (c) Fc(CH)_2_CO_2_H, (d) Fc(CH_2_)_2_CO_2_H, (e) Fc(CH_2_)_3_CO_2_H and (f) FcCO(CH_2_)_2_CO_2_H, at scan rates 0.100 (red) and 5.00 (black) Vs^−1^. Scans initiated in a positive direction. Data for the peak oxidation potential (E_pa_) and the formal reduction potential (E^0’^) of DmFc (internal standard, left) and the indicated ferrocene-containing carboxylic acid (right) are indicated in V.

This article presents redox data of six ferrocene-containing carboxylic acids, 1 – 6, reported versus the redox couple ferrocene (Fc) at 0, using decamethylferrocene (DmFc) as internal standard [2], see Figure 1 for the series of complexes of this data study. Cyclic voltammograms obtained in dichloromethane (DCM) and acetonitrile (ACN) for compound 1 – 6, with DmFc as internal standard, are shown in Figure 2–Figure 9. The cyclic voltammograms of DmFc and ferrocene in DCM and ACN are shown in Fig. 10, Fig. 11. Electrochemical data obtained from the cyclic voltammograms at scan rates 0.05 Vs^−1^ – 5.00 Vs^−1^ are tabulated in Table 1–Table 12 (0.10 Vs^−1^data from reference [1]). Presented data is related to the research article “Solvent and substituent effect on Electrochemistry of ferrocenylcarboxylic acids”, published in Journal of Electroanalytical Chemistry [1]. The electronic influence of the different carboxylic acids substituents on the redox potential of the ferrocenylgroup they are attached to, is illustrated in Figure 2 and Figure 3. The electronic influence of the electron-withdrawing carbonyl group on the iron's oxidation potential, depends on how close the carbonyl group is to the iron. Redox data of ferrocene-containing compounds are important for application in asymmetric catalysis [3], [4], [5], [6], energy transfer processes [7], biological applications [8,9], as additives in highburning rate composite rocket propellants [10] and non-linear optics [6].

**Fig. 4:**
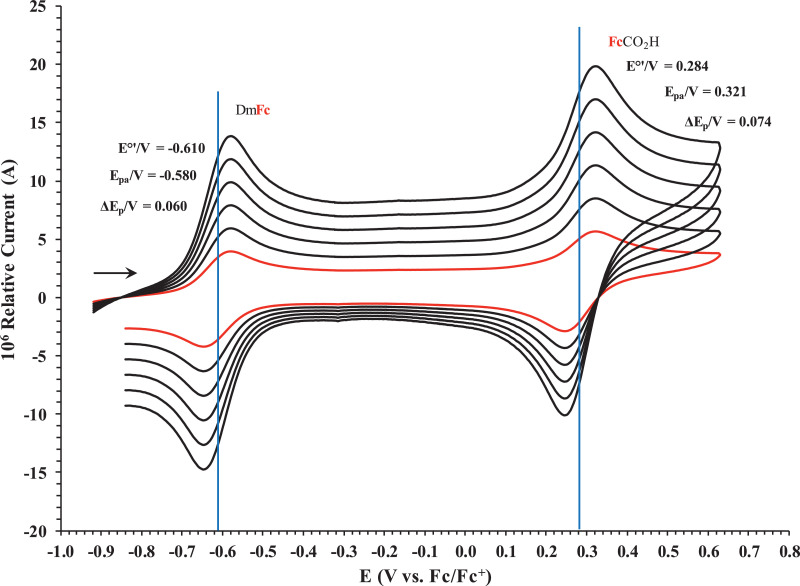
Cyclic voltammograms in DCM of FcCO_2_H at scan rates 0.050 (smallest peak currents), 0.100, 0.200, 0.300, 0.400 and 0.500 (largest peak currents) Vs^−1^. All scans initiated in a positive direction. Data for the peak oxidation potential (E_pa_), the formal reduction potential (E^0`^) and the peak current separation ΔE_p_ of the Fe^II/III^ oxidation of DmFc (internal standard, left) and the indicated ferrocene-containing carboxylic acid (right) are indicated in V.

**Fig. 5:**
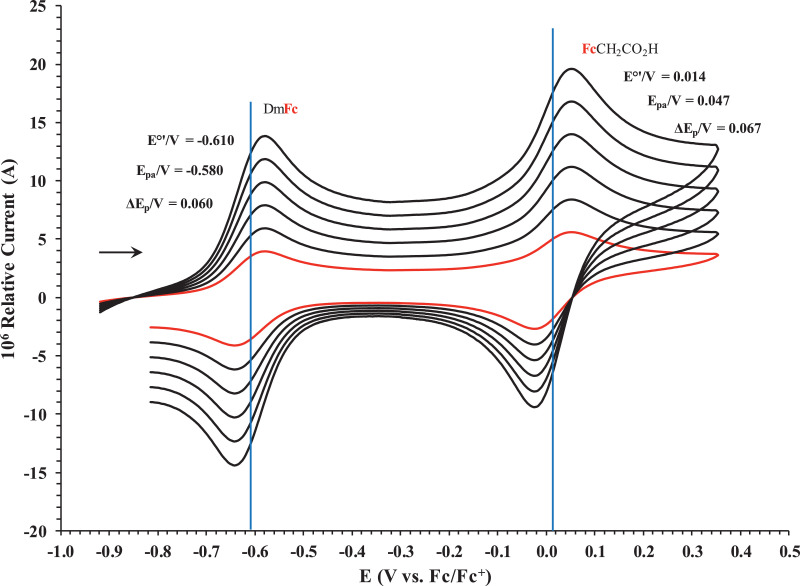
Cyclic voltammograms in DCM of FcCH_2_CO_2_H at scan rates 0.050 (smallest peak currents), 0.100, 0.200, 0.300, 0.400 and 0.500 (largest peak currents) Vs^−1^. All scans initiated in a positive direction. Data for the peak oxidation potential (E_pa_), the formal reduction potential (E^0`^) and the peak current separation ΔE_p_ of the Fe^II/III^ oxidation of DmFc (internal standard, left) and the indicated ferrocene-containing carboxylic acid (right) are indicated in V.

**Fig. 6:**
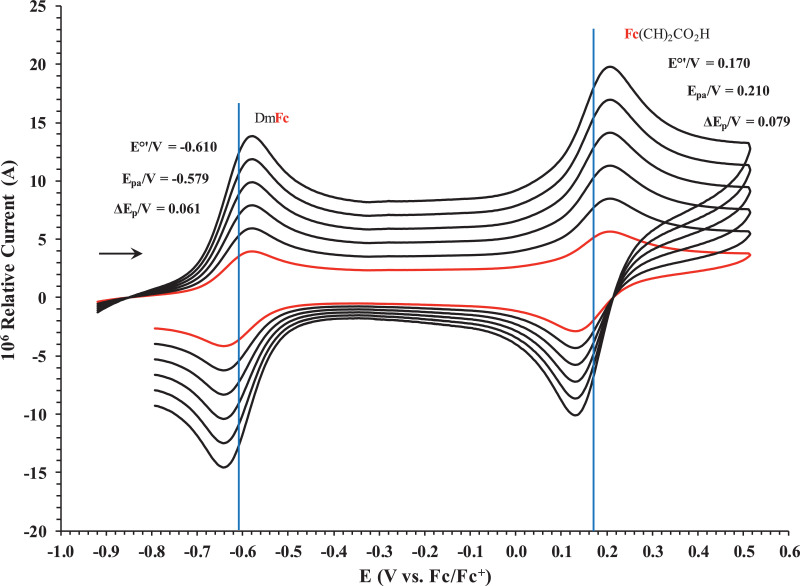
Cyclic voltammograms in DCM of Fc(CH)_2_CO_2_H at scan rates 0.050 (smallest peak currents), 0.100, 0.200, 0.300, 0.400 and 0.500 (largest peak currents) Vs^−1^. All scans initiated in a positive direction. Data for the peak oxidation potential (E_pa_), the formal reduction potential (E^0`^) and the peak current separation ΔE_p_ of the Fe^II/III^ oxidation of DmFc (internal standard, left) and the indicated ferrocene-containing carboxylic acid (right) are indicated in V.

**Fig. 7:**
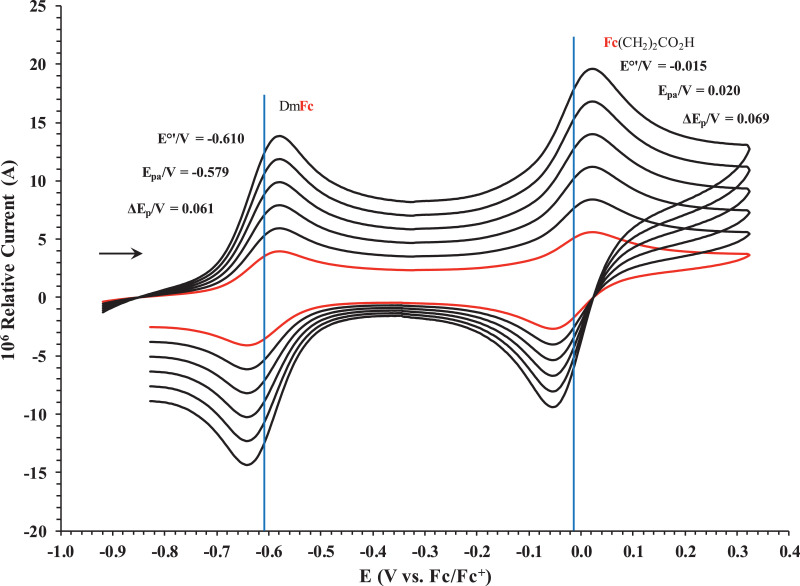
Cyclic voltammograms in DCM of Fc(CH_2_)_2_CO_2_H at scan rates 0.050 (smallest peak currents), 0.100, 0.200, 0.300, 0.400 and 0.500 (largest peak currents) Vs^−1^. All scans initiated in a positive direction. Data for the peak oxidation potential (E_pa_), the formal reduction potential (E^0`^) and the peak current separation ΔE_p_ of the Fe^II/III^ oxidation of DmFc (internal standard, left) and the indicated ferrocene-containing carboxylic acid (right) are indicated in V.

**Fig. 8:**
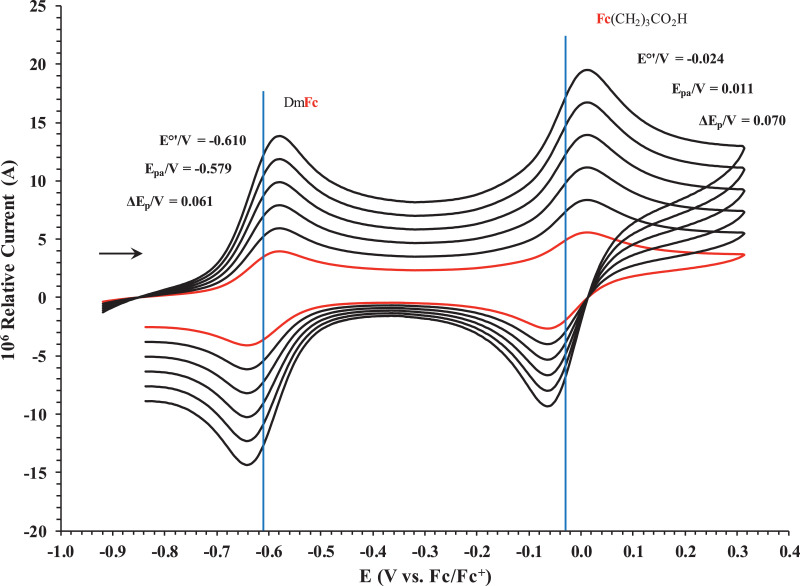
Cyclic voltammograms in DCM of Fc(CH_2_)_3_CO_2_H at scan rates 0.050 (smallest peak currents), 0.100, 0.200, 0.300, 0.400 and 0.500 (largest peak currents) Vs^−1^. All scans initiated in a positive direction. Data for the peak oxidation potential (E_pa_), the formal reduction potential (E^0`^) and the peak current separation ΔE_p_ of the Fe^II/III^ oxidation of DmFc (internal standard, left) and the indicated ferrocene-containing carboxylic acid (right) are indicated in V.

**Fig. 9:**
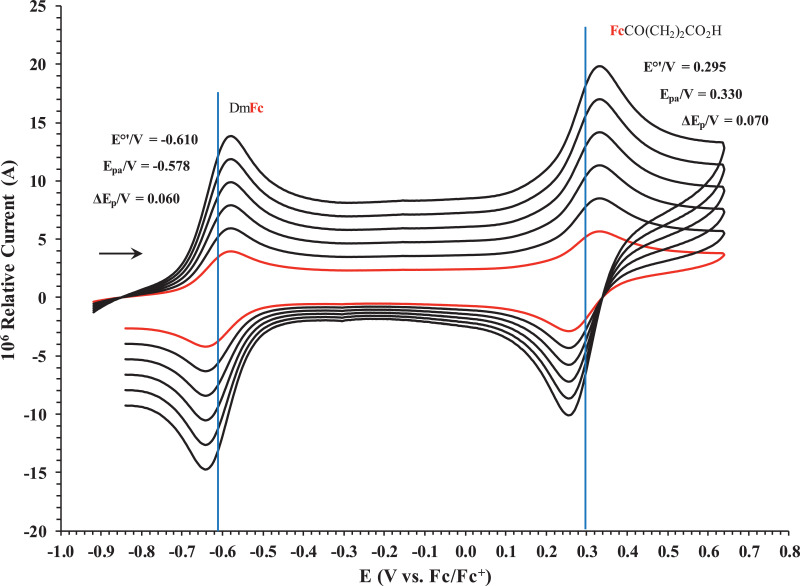
Cyclic voltammograms in DCM of FcCO(CH_2_)_2_CO_2_Hat scan rates 0.050 (smallest peak currents), 0.100, 0.200, 0.300, 0.400 and 0.500 (largest peak currents) Vs^−1^. All scans initiated in a positive direction. Data for the peak oxidation potential (E_pa_), the formal reduction potential (E^0`^) and the peak current separation ΔE_p_ of the Fe^II/III^ oxidation of DmFc (internal standard, left) and the indicated ferrocene-containing carboxylic acid (right) are indicated in V.

**Fig. 10:**
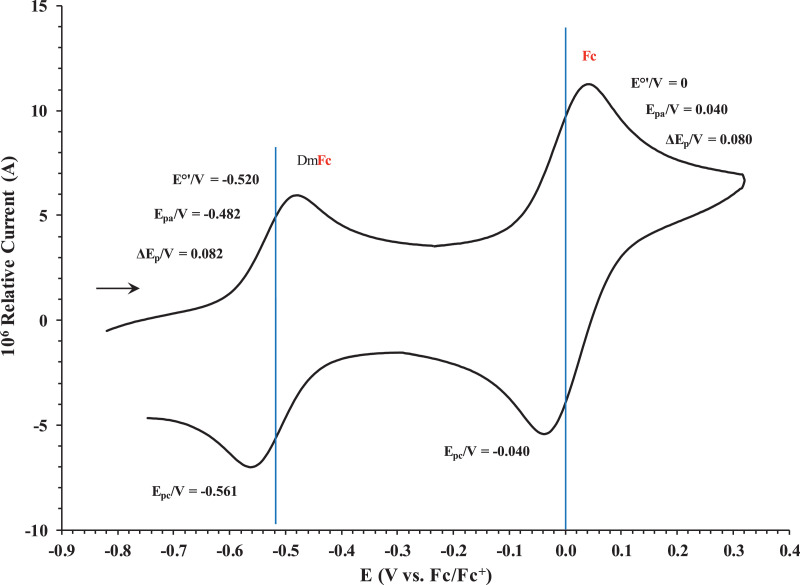
Cyclic voltammograms in ACN of decamethylferrocene and ferrocene at scan rate 0.100 Vs^−1^. The scan is initiated in a positive direction. Data for the peak oxidation potential (E_pa_), the formal reduction potential (E^0`^) and the peak current separation ΔE_p_ of the Fe^II/III^ oxidation of DmFc (internal standard, left) and Fc (right) is indicated in V.

**Fig. 11:**
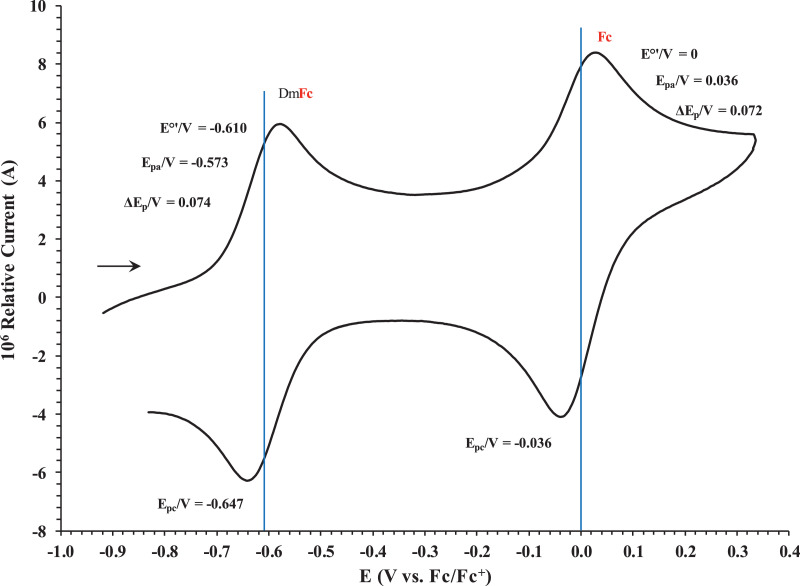
Cyclic voltammograms in DCM of decamethylferrocene and ferrocene at scan rate 0.100 Vs^−1^. The scan is initiated in a positive direction. Data for the peak oxidation potential (E_pa_), the formal reduction potential (E^0`^) and the peak current separation ΔE_p_ of the Fe^II/III^ oxidation of DmFc (internal standard, left) and Fc (right) is indicated in V.

**Table 1:** Electrochemical data (potential in V *vs* Fc/Fc^+^) in ACN for *c.a*. 5 × 10^−4^ mol dm^−3^ of FcCO_2_H at indicated scan rates (ν in V/s).

ν (V/s)	*E*_pa_ / V	Δ*E*_p_ / V	*E*^o′^ / V	*i*_pa_ / μA	*i*_pc_/*i*_pa_
DmFc					
**0.100**	**-0.480**	**0.080**	**-0.520**	**3.21**	**0.99**
FcCO_2_H					
0.050	0.217	0.078	0.178	2.16	0.99
**0.100**	**0.218**	**0.080**	**0.178**	**3.86**	**0.99**
0.200	0.218	0.080	0.178	5.54	0.99
0.300	0.218	0.080	0.178	6.59	0.99
0.400	0.219	0.082	0.178	8.01	0.99
0.500	0.219	0.082	0.178	9.04	0.99
5.000	0.220	0.084	0.178	26.91	0.99

**Table 2:** Electrochemical data (potential in V *vs* Fc/Fc^+^) in DCM for *c.a*. 5 × 10^−4^ mol dm^−3^ of FcCO_2_H at indicated scan rates (ν in V/s).

ν (V/s)	*E*_pa_ / V	Δ*E*_p_ / V	*E*^o′^ / V	*i*_pa_ / μA	*i*_pc_/*i*_pa_
DmFc					
**0.100**	**-0.580**	**0.060**	**-0.610**	**3.47**	**0.99**
FcCO_2_H					
0.050	0.320	0.072	0.284	2.33	0.99
**0.100**	**0.321**	**0.074**	**0.284**	**3.60**	**0.99**
0.200	0.321	0.074	0.284	5.62	0.99
0.300	0.321	0.074	0.284	6.80	0.99
0.400	0.322	0.076	0.284	8.16	0.99
0.500	0.322	0.076	0.284	9.14	0.99
5.000	0.323	0.078	0.284	25.27	0.99

**Table 3:** Electrochemical data (potential in V *vs* Fc/Fc^+^) in ACN for *c.a*. 5 × 10^−4^ mol dm^−3^ of FcCH_2_CO_2_H at indicated scan rates (ν in V/s).

ν (V/s)	*E*_pa_ / V	Δ*E*_p_ / V	*E*^o′^ / V	*i*_pa_ / μA	*i*_pc_/*i*_pa_
DmFc					
**0.100**	**-0.481**	**0.078**	**-0.520**	**3.35**	**0.99**
FcCH_2_CO_2_H					
0.050	-0.048	0.080	-0.088	2.31	0.99
**0.100**	**-0.047**	**0.082**	**-0.088**	**3.57**	**0.99**
0.200	-0.047	0.083	-0.088	4.29	0.99
0.300	-0.046	0.084	-0.088	6.45	0.99
0.400	-0.046	0.085	-0.088	8.15	0.99
0.500	-0.045	0.086	-0.088	10.22	0.99
5.000	-0.043	0.090	-0.088	27.12	0.99

**Table 4:** Electrochemical data (potential in V *vs* Fc/Fc^+^) in DCM for *c.a*. 5 × 10^−4^ mol dm^−3^ of FcCH_2_CO_2_H at indicated scan rates (ν in V/s).

ν (V/s)	*E*_pa_ / V	Δ*E*_p_ / V	*E*^o′^ / V	*i*_pa_ / μA	*i*_pc_/*i*_pa_
DmFc					
**0.100**	**-0.580**	**0.060**	**-0.610**	**3.65**	**0.99**
FcCH_2_CO_2_H					
0.050	0.046	0.065	0.014	2.21	0.99
**0.100**	**0.047**	**0.067**	**0.014**	**3.78**	**0.99**
0.200	0.047	0.068	0.014	4.35	0.99
0.300	0.047	0.068	0.014	6.25	0.99
0.400	0.047	0.069	0.014	8.24	0.99
0.500	0.048	0.070	0.014	10.51	0.99
5.000	0.049	0.072	0.014	25.72	0.99

**Table 5:** Electrochemical data (potential in V *vs* Fc/Fc^+^) in ACN for *c.a*. 5 × 10^−4^ mol dm^−3^ of Fc(CH)_2_CO_2_H at indicated scan rates (ν in V/s).

ν (V/s)	*E*_pa_ / V	Δ*E*_p_ / V	*E*^o′^ / V	*i*_pa_ / μA	*i*_pc_/*i*_pa_
DmFc					
**0.100**	**-0.480**	**0.080**	**-0.520**	**3.82**	**0.99**
Fc(CH)_2_CO_2_H					
0.050	0.104	0.076	0.066	2.01	0.99
**0.100**	**0.105**	**0.078**	**0.066**	**3.93**	**0.99**
0.200	0.105	0.078	0.066	4.84	0.99
0.300	0.105	0.078	0.066	6.48	0.99
0.400	0.106	0.080	0.066	7.85	0.99
0.500	0.106	0.080	0.066	8.84	0.99
5.000	0.107	0.082	0.066	26.87	0.99

**Table 6:** Electrochemical data (potential in V *vs* Fc/Fc^+^) in DCM for *c.a*. 5 × 10^−4^ mol dm^−3^ of Fc(CH)_2_CO_2_H at indicated scan rates (ν in V/s).

ν (V/s)	*E*_pa_ / V	Δ*E*_p_ / V	*E*^o′^ / V	*i*_pa_ / μA	*i*_pc_/*i*_pa_
DmFc					
**0.100**	**-0.579**	**0.061**	**-0.610**	**3.25**	**0.99**
Fc(CH)_2_CO_2_H					
0.050	0.209	0.078	0.170	1.98	0.99
**0.100**	**0.210**	**0.079**	**0.170**	**3.36**	**0.99**
0.200	0.210	0.079	0.170	4.91	0.99
0.300	0.210	0.079	0.170	6.54	0.99
0.400	0.211	0.080	0.170	7.94	0.99
0.500	0.211	0.080	0.170	8.94	0.99
5.000	0.212	0.084	0.170	25.58	0.99

**Table 7:** Electrochemical data (potential in V *vs* Fc/Fc^+^) in ACN for *c.a*. 5 × 10^−4^ mol dm^−3^ of Fc(CH_2_)_2_CO_2_H at indicated scan rates (ν in V/s).

ν (V/s)	*E*_pa_ / V	Δ*E*_p_ / V	*E*^o′^ / V	*i*_pa_ / μA	*i*_pc_/*i*_pa_
DmFc					
**0.100**	**-0.482**	**0.076**	**-0.520**	**3.58**	**0.99**
Fc(CH_2_)_2_CO_2_H					
0.050	-0.078	0.070	-0.113	2.22	0.99
**0.100**	**-0.077**	**0.072**	**-0.113**	**3.74**	**0.99**
0.200	-0.077	0.073	-0.113	4.84	0.99
0.300	-0.076	0.074	-0.113	6.48	0.99
0.400	-0.076	0.075	-0.113	8.39	0.99
0.500	-0.075	0.076	-0.113	9.55	0.99
5.000	-0.074	0.078	-0.113	26.71	0.99

**Table 8:** Electrochemical data (potential in V *vs* Fc/Fc^+^) in DCM for *c.a*. 5 × 10^−4^ mol dm^−3^ of Fc(CH_2_)_2_CO_2_H at indicated scan rates (ν in V/s).

ν (V/s)	*E*_pa_ / V	Δ*E*_p_ / V	*E*^o′^ / V	*i*_pa_ / μA	*i*_pc_/*i*_pa_
DmFc					
**0.100**	**-0.579**	**0.060**	**-0.610**	**3.74**	**0.99**
Fc(CH_2_)_2_CO_2_H					
0.050	0.019	0.068	-0.015	2.12	0.99
**0.100**	**0.020**	**0.070**	**-0.015**	**3.87**	**0.99**
0.200	0.020	0.070	-0.015	5.11	0.99
0.300	0.020	0.070	-0.015	6.73	0.99
0.400	0.021	0.072	-0.015	8.01	0.99
0.500	0.021	0.072	-0.015	9.11	0.99
5.000	0.022	0.074	-0.015	25.32	0.99

**Table 9:** Electrochemical data (potential in V *vs* Fc/Fc^+^) in ACN for *c.a*. 5 × 10^−4^ mol dm^−3^ of Fc(CH_2_)_3_CO_2_H at indicated scan rates (ν in V/s).

ν (V/s)	*E*_pa_ / V	Δ*E*_p_ / V	*E*^o′^ / V	*i*_pa_ / μA	*i*_pc_/*i*_pa_
DmFc					
**0.100**	**-0.482**	**0.075**	**-0.520**	**3.64**	**0.99**
Fc(CH_2_)_3_CO_2_H					
0.050	-0.091	0.077	-0.131	2.52	0.99
**0.100**	**-0.092**	**0.078**	**-0.131**	**3.83**	**0.99**
0.200	-0.092	0.079	-0.131	5.15	0.99
0.300	-0.092	0.080	-0.131	6.95	0.99
0.400	-0.093	0.081	-0.131	8.35	0.99
0.500	-0.093	0.082	-0.131	9.69	0.99
5.000	-0.094	0.084	-0.131	27.31	0.99

**Table 10:** Electrochemical data (potential in V *vs* Fc/Fc^+^) in DCM for *c.a*. 5 × 10^−4^ mol dm^−3^ of Fc(CH_2_)_3_CO_2_H at indicated scan rates (ν in V/s).

ν (V/s)	*E*_pa_ / V	Δ*E*_p_ / V	*E*^o′^ / V	*i*_pa_ / μA	*i*_pc_/*i*_pa_
DmFc					
**0.100**	**-0.579**	**-0.061**	**-0.610**	**3.89**	**0.99**
Fc(CH_2_)_3_CO_2_H					
0.050	0.010	0.068	-0.024	2.39	0.99
**0.100**	**0.011**	**0.070**	**-0.024**	**3.98**	**0.99**
0.200	0.011	0.070	-0.024	5.26	0.99
0.300	0.012	0.072	-0.024	6.82	0.99
0.400	0.012	0.072	-0.024	8.23	0.99
0.500	0.013	0.074	-0.024	9.46	0.99
5.000	0.014	0.076	-0.024	25.04	0.99

**Table 11:** Electrochemical data (potential in V *vs* Fc/Fc^+^) in ACN for *c.a*. 5 × 10^−4^ mol dm^−3^ of FcCO(CH_2_)_2_CO_2_H at indicated scan rates (ν in V/s).

ν (V/s)	*E*_pa_ / V	Δ*E*_p_ / V	*E*^o′^ / V	*i*_pa_ / μA	*i*_pc_/*i*_pa_
DmFc					
**0.100**	**-0.480**	**0.080**	**-0.520**	**3.13**	**0.99**
FcCO(CH_2_)_2_CO_2_H					
0.050	0.219	0.078	0.180	2.31	0.99
**0.100**	**0.220**	**0.080**	**0.180**	**3.52**	**0.99**
0.200	0.220	0.080	0.180	5.29	0.99
0.300	0.220	0.080	0.180	6.85	0.99
0.400	0.221	0.082	0.180	8.66	0.99
0.500	0.221	0.082	0.180	9.85	0.99
5.000	0.222	0.084	0.180	27.26	0.99

**Table 12:** Electrochemical data (potential in V *vs* Fc/Fc^+^) in DCM for *c.a*. 5 × 10^−4^ mol dm^−3^ of FcCO(CH_2_)_2_CO_2_H at indicated scan rates (ν in V/s).

ν (V/s)	*E*_pa_ / V	Δ*E*_p_ / V	*E*^o′^ / V	*i*_pa_ / μA	*i*_pc_/*i*_pa_
DmFc					
**0.100**	**-0.580**	**0.060**	**-0.610**	**3.51**	**0.99**
FcCO(CH_2_)_2_CO_2_H					
0.050	0.329	0.068	0.295	2.42	0.99
**0.100**	**0.330**	**0.070**	**0.295**	**3.67**	**0.99**
0.200	0.330	0.070	0.295	5.22	0.99
0.300	0.331	0.072	0.295	6.99	0.99
0.400	0.331	0.072	0.295	8.31	0.99
0.500	0.332	0.074	0.295	9.55	0.99
5.000	0.335	0.080	0.295	25.84	0.99

## 2 Experimental Design, Materials, and Methods

Electrochemical studies through cyclic voltammetry (CV) experiments were performed in an M Bruan Lab Master SP glove box under a high purity argon atmosphere (H_2_O and O_2_< 10 ppm), utilising a Princeton Applied Research PARSTAT 2273 potentiostat running Powersuite software (Version 2.58). The cyclic voltammetry experimental setup consists of a cell with three electrodes, namely (i) a glassy carbon electrode as working electrode, (ii) a platinum wire auxiliary and (ii) a platinum wire as pseudo reference electrode. The glassy carbon working electrode was polished and prepared before every experiment on a Buhler polishing mat first with 1-micron and then with ¼-micron diamond paste, rinsed with H_2_O, acetone and DCM, and dried before each experiment. The electrochemical analysis is performed in dichloromethane (DCM, anhydrous, ≥ 99.8%, containing 40-150 ppm amylene as a stabilizer) and in acetonitrile (ACN, anhydrous, 99.8%) as solvents, at RT. Solutions were made in 0.001 dm^3^ spectrochemical grade anhydrous DCM or ACN containing ca. 5 × 10^−4^ M of analyte, 5 × 10^−4^ mol dm^−3^ of internal reference (decamethylferrocene, DmFc) and 0.1 mol dm^−3^ of supporting electrolyte tetrabutylammonium tetrakispentafluorophenylborate, [N(*^n^*Bu)_4_][B(C_6_F_5_)_4_] in DCM, or tetrabutylammonium hexafluorophosphate, TBAPF_6_, [N(*^n^*Bu)_4_][PF_6_] in ACN. Experimental potential data was measured *vs.* the redox couple of decamethylferrocene DmFc as internal standard [2] and reported vs. the redox couple of ferrocene, Fc, as suggested by IUPAC [11]. E°`(DmFc) = -0.610 V vs. Fc/Fc^+^ at 0 V in DCM/[N(Bu)_4_][B(C_6_F_5_)_4_] and -0.520 vs. Fc/Fc^+^ at 0 V in ACN/[N(Bu)_4_][PF_6_]. Scan rates were between 0.05 and 5.00 Vs^−1^.

## Declaration of Competing Interest

The authors declare that they have no known competing financial interests or personal relationships which have, or could be perceived to have, influenced the work reported in this article.

## Appendix Supplementary materials

Image, application 1
